# Validating genetic markers of response to recombinant human growth hormone in children with growth hormone deficiency and Turner syndrome: the PREDICT validation study

**DOI:** 10.1530/EJE-16-0357

**Published:** 2016-10-19

**Authors:** Adam Stevens, Philip Murray, Jerome Wojcik, John Raelson, Ekaterina Koledova, Pierre Chatelain, Peter Clayton, The PREDICT Investigator Group

**Affiliations:** 1 Faculty of BiologyMedicine and Health, University of Manchester and Manchester Academic Health Science Centre, Royal Manchester Children’s Hospital, Central Manchester University Hospitals NHS Foundation Trust, Manchester, UK; 2 Quartz BioGeneva, Switzerland; 3 Genizon BioSciencesSt Laurent, Quebec, Canada; 4 Merck KGaADarmstadt, Germany; 5 Department PediatrieHôpital Mère-Enfant – Université Claude Bernard, Lyon, France

## Abstract

**Objective:**

Single-nucleotide polymorphisms (SNPs) associated with the response to recombinant human growth hormone (r-hGH) have previously been identified in growth hormone deficiency (GHD) and Turner syndrome (TS) children in the PREDICT long-term follow-up (LTFU) study (Nbib699855). Here, we describe the PREDICT validation (VAL) study (Nbib1419249), which aimed to confirm these genetic associations.

**Design and methods:**

Children with GHD (*n* = 293) or TS (*n* = 132) were recruited retrospectively from 29 sites in nine countries. All children had completed 1 year of r-hGH therapy. 48 SNPs previously identified as associated with first year growth response to r-hGH were genotyped. Regression analysis was used to assess the association between genotype and growth response using clinical/auxological variables as covariates. Further analysis was undertaken using random forest classification.

**Results:**

The children were younger, and the growth response was higher in VAL study. Direct genotype analysis did not replicate what was found in the LTFU study. However, using exploratory regression models with covariates, a consistent relationship with growth response in both VAL and LTFU was shown for four genes – *SOS1* and *INPPL1* in GHD and *ESR1* and *PTPN1* in TS. The random forest analysis demonstrated that only clinical covariates were important in the prediction of growth response in mild GHD (>4 to <10 μg/L on GH stimulation test), however, in severe GHD (≤4 μg/L) several SNPs contributed (in *IGF2*, *GRB10*, *FOS*, *IGFBP3* and *GHRHR*).

**Conclusions:**

The PREDICT validation study supports, in an independent cohort, the association of four of 48 genetic markers with growth response to r-hGH treatment in both pre-pubertal GHD and TS children after controlling for clinical/auxological covariates. However, the contribution of these SNPs in a prediction model of first-year response is not sufficient for routine clinical use.

## Introduction

Although therapy with recombinant human growth hormone (r-hGH) is efficacious, there is substantial interindividual and interdisease variability in growth response to r-hGH therapy (1, 2, 3). Methods that predict growth response to r-hGH have been developed based on statistical models using baseline auxological and biochemical parameters (4, 5, 6, 7).

Genetic polymorphisms associated with response to r-hGH therapy are recognised, with the most extensively studied being the exon 3 *GHR* deletion (GHR-D3) (8). The possibility of using genetic markers in the prediction of response to r-hGH therapy, thus allowing early personalised dose optimisation (9), formed the underlying rationale for the PREDICT study designed to assess pharmacogenomic relationships with response to r-hGH in GHD and TS. This study had two components: (i) a phase IV, open-label, prospective multicentre study using GH-treatment-naïve children with GHD and TS over the first month of r-hGH treatment (Nbib256126) and (ii) a long-term follow-up (LTFU), which was an observational 5-year study collecting routine clinical and auxological information at the child’s standard annual visits during r-hGH treatment (Nbib699855). The association of genetic markers (single-nucleotide polymorphisms (SNPs)) within selected candidate genes (related to the GH/IGF1 axis, cell signalling and metabolism) with change in biomarkers (e.g. serum IGF-I) over 1 month and growth response over 1 year have been reported (10, 11). The genetic associations with height velocity at year one of treatment with r-hGH implicated eleven genes in GHD and ten in TS (11). Different sets of SNPs were found to be related to the two conditions implying that genetic influence on growth response to r-hGH is disease specific (11).

Growth prediction models for the main growth disorders treated with r-hGH (4, 6, 12, 13) have been developed by the KIGS pharmacoepidemiological survey (Pfizer International Growth Study) (4). These statistical models provide the clinician with the ability to generate individualised data on short- and mid-term growth, which can be used for counselling, adjusting GH dosing (14) or categorising response after at least 1 year of treatment. The variability of growth response in the first year of treatment explained by the KIGS models has been calculated as 61 and 46% for GHD (4) and TS (5) respectively. These models have not incorporated any direct genetic information although surrogate genetic markers, such as parental heights are used. The relationship between mid-parental height and the final height of offspring has been shown to explain 40% of the sex- and age-adjusted height variance in normal growth (15).

The use of genomic data to facilitate the prediction of growth response must account for the influence of many factors. Human gene expression has been shown to vary in response to the phase of physical development (infancy, childhood and puberty) implying that gene ontogeny is a factor in growth response (16). Within the PREDICT study, gene and environment interactions have been defined in children with GHD among SNP carriage, location where treatment was given (as measured by summer daylight exposure at that location) and first-year response to r-hGH (17). An interaction between GHD severity and the carriage of GHR-D3 has also been determined in the PREDICT study (18). Together these data support the need to consider the interaction factors in genomic analyses.

The identification of ‘good’ and ‘poor’ responders to therapy represents both a major clinical need and a challenge; for example, a child who is predicted to respond poorly may need to start r-hGH on a higher than average dose for that condition or should not be treated at all, whereas for a good responder, the dose can be reduced, avoiding exposure to supra-physiological IGF-I concentrations and reducing the overall cost of r-hGH therapy per patient.

The aim of this study was to validate the SNPs associated in the PREDICT LTFU study with growth response to r-hGH therapy in a second cohort of children with either GHD or TS. A secondary aim was to investigate potential gene × environment interactions within the data.

## Methods

### Study design

The PREDICT validation study (Nbib1419249) was planned as a retrospective replication analysis of 48 previously identified associations between SNPs and GH response (11). Two hundred and ninety three children with GHD and 132 children with TS were recruited from 29 sites in nine countries. Three clinical endpoints – (i) change in height (cm); (ii) change in height SDS and (iii) height velocity SDS after 1 year of treatment – were categorised by quartiles such as (L)ow (Q1), (I)ntermediate (Q2 and Q3) and (H)igh (Q4) response for each patient, stratified by gender and by age group (<8 years, 8–12 years and >12 years). The study was powered to validate at least one SNP.

All patients were recruited through their local growth clinics; they were pre-pubertal when GH treatment was started. The diagnosis of GHD was based on two different stimulation tests with a peak GH < 10 µg/L, and patients were selected for r-hGH treatment by their local units. Subjects were excluded with acquired GHD due to central nervous system disorders, such as tumour, trauma, infection, infiltration, irradiation and cranial surgery. Other hormone deficiencies (cortisol and thyroxine), if present, were appropriately treated. The median peak GH value was 5.2 μg/L (Table 1). R-hGH was administered subcutaneously, once daily at bedtime.

**Table 1:** Demographic characteristics of the study populations at the start of r-hGH therapy. Data are presented as number (%) or median (minimum, maximum).

	**VAL GHD** (*n* = 293)	**LTFU GHD** (*n* = 115)^†^	**VAL TS** (*n* = 132)	**LTFU TS** (*n* = 67)^†^
Male (%)	208 (71)*	69 (60)*	–	–
Female (%)	85 (29)*	46 (40)*	132 (100)*	67 (100)*
Age (years)	6.2** (0.4, 16.3)	9.8 (2, 15)	5.8** (1.1, 14.4)	9.1 (3, 16)
Height SDS	−2.5 (−7.2, −0.1)	−2.1 (−6.5, −0.3)	−2.2 (−5.9, 0.6)	−2.4 (−5.4, −0.2)
MPH SDS	−0.3** (−3.3, 3.1)	−0.8 (−4, +2)	0.3** (−2.5, 3.6)	−0.1 (−4, +2)
DTH SDS***	−2.3** (7.3, 1.1)	−1.3 (−5.7, 3.3)	−2.4** (6.5, 0.3)	−2.2 (−7.8, 1.1)
GH dose (mg/kg.day)	0.026** (0.02, 0.05)	0.034 (0.01, 0.14)	0.046** (0.02, 0.08)	0.050 (0.01, 0.9)
GH peak (μg/L)	5.2** (0, 10)	4.1 (0, 9)	–	–

*All were Tanner Stage 1 at baseline; **P value ≤0.05 (*t*-test across studies); ***DTH SDS defined as height SDS at baseline – MPH SDS; ^†^Numbers taken from Clayton *et al*. (11).

BMI, body mass index; DTH, distance to target height; GH, growth hormone; GHD, growth hormone deficiency; MPH, mid-parental height; Q, quartile; SDS, standard deviation score.

This study was conducted in compliance with ethical principles based on the Declaration of Helsinki, the International Conference on Harmonization Tripartite Guideline for Good Clinical Practice and all applicable regulatory requirements.

### Genetic analysis

Genotyping was performed centrally on DNA extracted from whole blood taken during a routine clinic visit before starting r-hGH therapy, using TaqMan probes (Life Technologies). All SNPs were checked for the proportion of missing data (<5%), the presence of Hardy–Weinberg equilibrium (HWE, *P* value >0.05 after multiple-testing correction) and minor allele frequency (MAF ≥10%).

### Statistical analysis

First, as planned per protocol, all associations were tested by categorical analysis to investigate the basal association with growth endpoint categories and thus replicate at least one of the 48 genetic associations found in LTFU (11). This was performed using year 1 quartiles for growth response categorisation and an exact Fisher test on 2 × 2 contingency tables. Both SNPs and growth endpoints were classified into two categories (Supplementary Table 1, see section on supplementary data given at the end of this article): dominant or recessive models for SNPS, and ‘low’ (<Q1 first quartile) vs ‘intermediate + high’ (≥Q1) or ‘high’ (≥Q3 third quartile) vs ‘low + intermediate’ (<Q3). Obtained *P* values were adjusted for multiple testing using a Benjamini–Hochberg correction based on the number of tested SNPs per disease (22 in GHD and 26 in TS) (Supplementary Table 1).

Second, a continuous association analysis on growth response was performed using the Kruskal–Wallis association test and three genetic models for each SNP coded AA/AB/BB, A being the major allele: genotypic (AA vs AB vs BB), dominant ((AA or AB) vs BB) and recessive (AA vs (AB or BB)).

Third, to account for differences between the LTFU and VAL cohorts, regression analysis was performed using models including gender (GHD), age, GH peak (GHD), mid-parental height SDS, distance to target height SDS (defined as (height SDS at baseline − mid-parental height SDS)), GH dose (average daily dose (mg/kg) by body weight) as covariates (Table 1), and interactions with and without the SNPs. A total of 729 and 81 models were tested respectively in GHD and TS. For each SNP, the model with the best *SNP × covariate* interaction term *P* value or the best *SNP* term *P* value was selected. This step accounted for the presence of gene interactions with patient variables.

Finally, a machine-learning approach, random forest classification (RFC) using 1000 trees, was used to predict growth endpoints (categorised into binary variables above and below the year 1 median) based on different combinations of auxological parameters and SNPs (Supplementary Table 1). The conditional importance was used to identify variables that contributed the most to the predictions. The predictions were assessed based on the accuracy (the sum of the true positives plus the sum of the true negatives divided by the total population) and area under the curve (AUC) of the receiver-operating characteristic (ROC) (the probability that a classifier will rank a randomly chosen positive observation higher than a randomly chosen negative observation). The 95% confidence interval of the AUC was computed with 1000 stratified bootstrap replicates. A *Z*-score was used to compare the analysed AUC with a value of 50%, and the *P* value was reported. This approach was chosen to provide confirmation of observations from the regression analyses and to negate the need for significant numbers of regression models. In addition, RFC effectively accounts for co-linearity between variables and thereby generates a hierarchy of importance for variables.

Throughout the validation of SNP association, Y1 and VAL were used as individual datasets and as a combined dataset. For the random forest classification of GHD severity and SNP association, analysis was performed on the combined data. The RFC analysis was performed with three different sets of variables: auxological parameters only, SNPs only and auxological parameters and SNPs together.

All statistical analyses were performed using R 3.2.2.

## Results

### Auxology

The demographic characteristics of the study population are shown in Table 1. The demographics of the PREDICT LTFU have been previously published (11).

**Figure 1:**
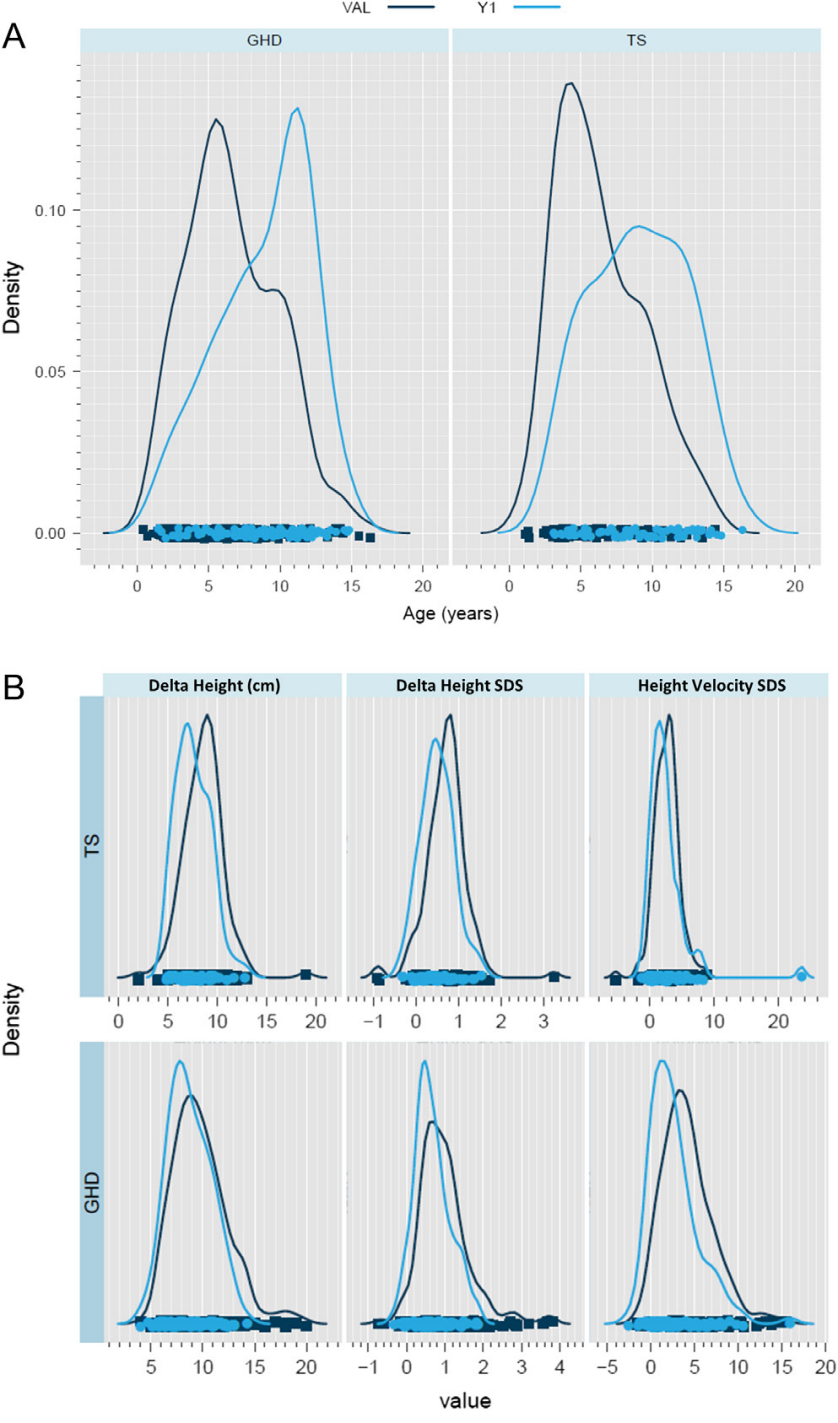
Comparison of demographics and growth end points between the PREDICT LTFU study and the VAL study. (A) Age distribution in GHD and TS children. (B) Growth response end point distribution after 1 year of treatment with r-hGH in GHD and TS children. Growth response end points used were, change in height (cm), change in height (SDS) and height velocity SDS.

There were no differences in gender distribution and SNP allele frequencies between the LTFU and VAL studies (data not shown). The demographics and baseline clinical data for the children included in the PREDICT LTFU study differed from those included in the VAL study. Age was lower (Fig. 1A) and growth responses were higher (Fig. 1B) in the VAL study compared with those in the LTFU study. GH peak (GHD: *P* ≤ 6 × 10^−5^) and mid-parental height SDS (GHD: *P* ≤ 2 × 10^−7^, TS: *P* ≤ 3 × 10^−3^) were higher in the VAL study. Distance below target height SDS was greater in the VAL study in GHD (*P* ≤ 2 × 10^−9^) and GH dose was lower in the VAL study (GHD: *P* ≤ 4 × 10^−21^, TS: *P* ≤ 6 × 10^−4^) (Table 1).

There was no genetic or gender bias between studies (data not shown).

### Categorical analysis of genetic associations with growth response endpoints

Within the VAL study, categorical analysis found no SNPs within genes that were significant using the year 1 quartiles. Two SNPs were significant in GHD before correction for multiple testing (*P* < 0.05). The rs3213221 SNP within *IGF2* was associated with both change in height (cm) and change in height SDS (CC genotype). The rs2267723 SNP within *GHRHR* was associated with height velocity SDS (carriage of the A allele).

### Continuous analysis of genetic associations with growth response endpoints

Continuous analysis revealed a set of significant associations with growth response endpoints (*P* < 0.05) (Table 2). No association withstood multiple-testing correction.

**Table 2:** Association of SNP markers with growth endpoints as continuous variables.

**Disease**	**Endpoint**	**Gene** (SNP)	**Marker** (response)	***P* value**		
				Dominant	Genotypic	Recessive
GHD	Change in height SDS	SOS1 (rs2888586)	TT (high)	0.111	0.0507	0.0225*
	Change in height (cm)	SOS1 (rs2888586)	TT (high)	0.2142	0.0795	0.0287*
TS		RB1 (rs9568036)	A (high)	0.5783	0.0219*	0.0222*
	Change in height (cm)	SOS1 (rs2168043)	CC (high)	0.0140*	0.0402*	0.1990
	HV SDS	IRS4 (rs2073115)	CC (high)	0.0414*	0.0861	0.2951
		RB1 (rs9568036)	GG (low)	0.9052	0.0654	0.0350*

* *P* value ≤0.05 (Kruskal–Wallis association test) not corrected for multiple testing.

### Regression modelling of the interaction between genetic markers of growth response and patient variables

In view of the significant difference in demographics and baseline clinical data, we performed the regression analysis (first-year growth response) in the VAL, LTFU and the combined datasets to identify consistent observations in modelling the effects of covariates.

Using regression modelling, multiple testing and overfitting were of concern (81 regression models analysed for TS and 729 for GHD). We therefore identified for both GHD and TS terms in the regression models common to one or more of the datasets (LFTU, VAL and combined datasets) using each of Δ height SDS, Δ height velocity SDS and Δ height in cm as outcomes. Visual inspections of associated genes per study were performed, and genes showing conflicting associations, e.g. in opposite direction, were discarded.

**Figure 2:**
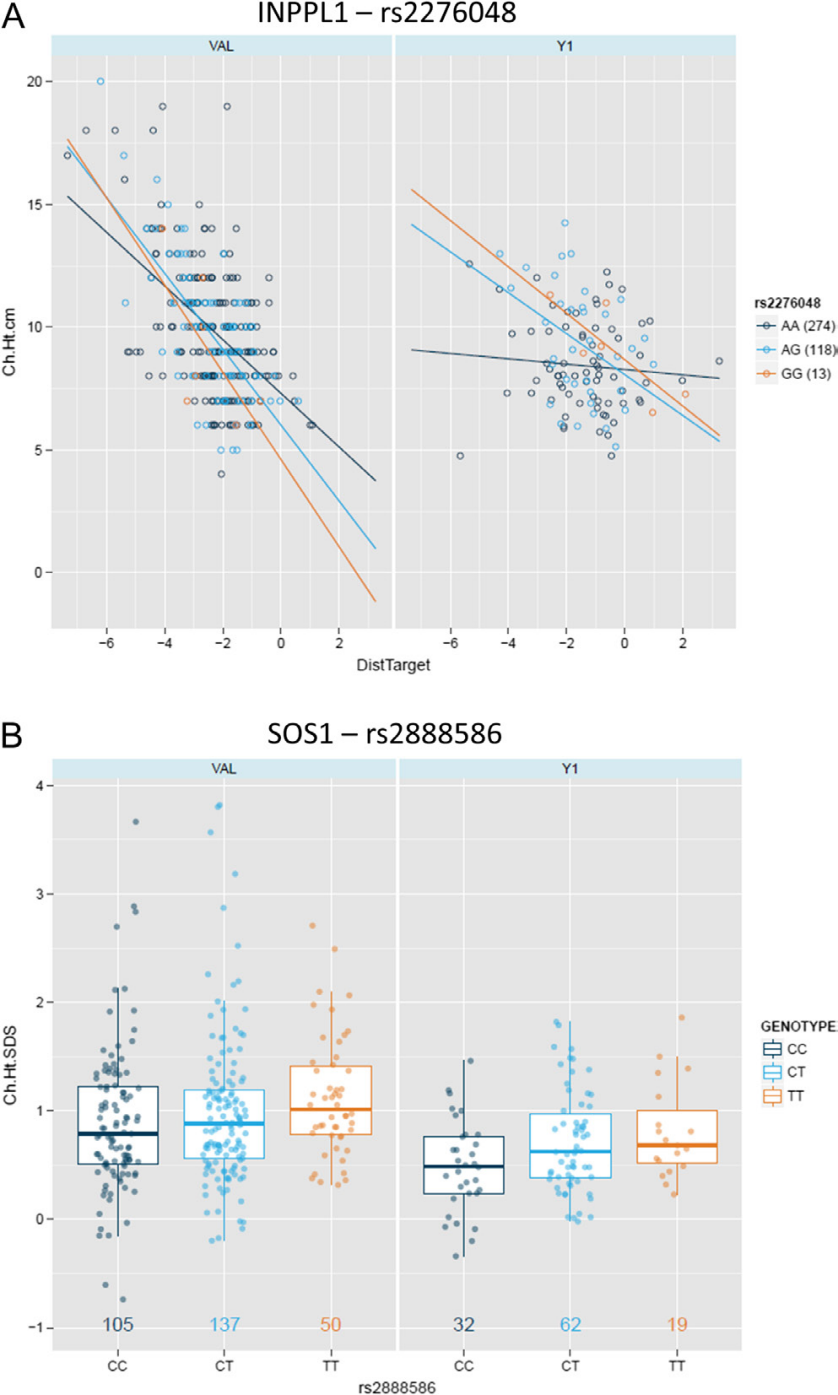
Replicated genetic associations in GHD. (A) Regression of mid-parental height and growth response (change in height (cm)) in the three genotypes of rs2276048 in both the Y1 of the PREDICT LTFU and the VAL studies (numbers of patients in brackets). (B) Box and whisker plots (median ± interquartile range) of growth response (change in height SDS) by genotype of rs2888586 in both the Y1 of the PREDICT LTFU and the VAL studies (numbers of patients (genotype colour)).

Terms consistently present in regression models of the three datasets for GHD children included SNPs within the *INPPL1* and *SOS1* genes. *INPPL1* (rs2276048) was associated with change in height (cm) in interaction with distance to target height SDS: The negative correlation between distance to target height and growth (change in height (cm)) is decreased in the carriers of the AA genotype of *INPPL1* ((rs2276048), *P* = 0.0057 VAL; *P* = 0.0144 LTFU)) (Fig. 2A). *SOS1* (rs2888586) is associated with change in height (SDS and cm) in GHD alone and in interaction with GHD severity (GH peak): the T allele is associated with better outcome (*P* = 0.0036 VAL; 6.4 × 10^−5^ LTFU) (Fig. 2B). *GRB10* (rs10248619) was associated with change in height (cm) in interaction with gender; however, the influence of genotype on response was not consistent between studies (Supplementary Fig. 1A). *IGFBP3* (rs3110697) was associated with height velocity SDS, but the influence of genotype on response was also not consistent between studies (Supplementary Fig. 1B). *CYP19A1* (rs10459592) was associated with height velocity SDS in interaction with gender but again, the influence of genotype on response was not consistent between studies (data not shown).

**Figure 3:**
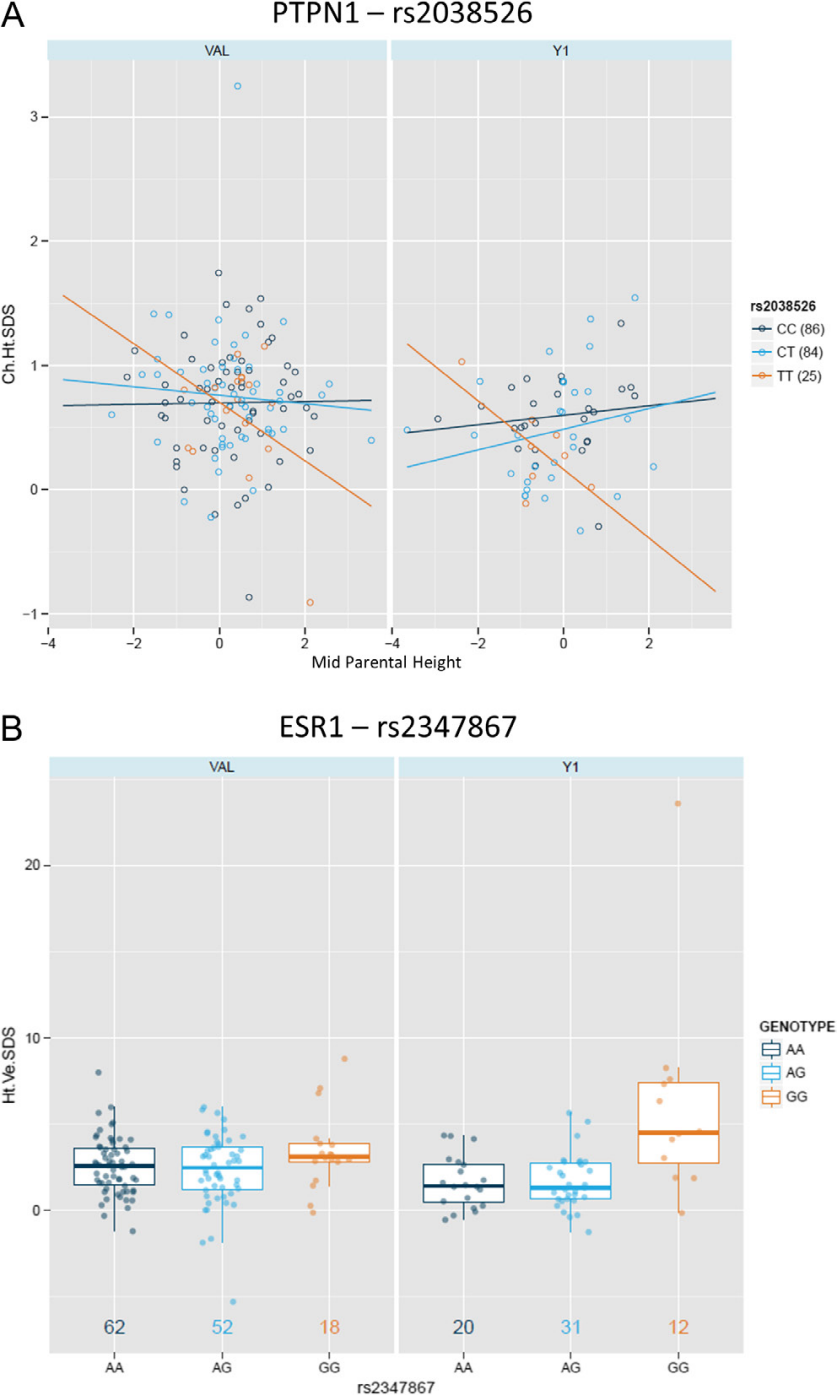
Replicated genetic associations in TS using regression modelling. (A) Regression of distance to target height and growth response (change in height SDS) in the three genotypes of rs2038526 in both the Y1 of the PREDICT LTFU and the VAL studies (numbers of patients in brackets). (B) Box and whisker plots (median ± interquartile range) of growth response (height velocity SDS) by genotype of rs2347867 in both the Y1 of the PREDICT LTFU and the VAL studies (numbers of patients (genotype colour)).

Terms consistently present in regression models for TS children included SNPs within the *PTPN1* and *ESR1* genes. SNP rs2038526 (*PTPN1*) was associated with change in height SDS in TS in interaction with mid-parental height SDS; the correlation between growth and mid-parental height SDS was strongly negative in TT carriers compared with close to null in non-carriers (*P* = 0.0113 VAL; 0.0055 LTFU) (Fig. 3A). SNP rs2347867 (*ESR1*) was associated with height velocity SDS in TS; the GG genotype was associated with better outcome (*P* = 0.0304 VAL; 6.2 × 10^−6^ LTFU) (Fig. 3B). *LHX4* (rs3845395) was associated with change in height (cm) in interaction with GH dose; however, the influence of genotype on response was not consistent between studies (data not shown).

In summary, analysis of the regression models provided modest validation of *INPPL1* (rs2276048) in GHD and *ESR1* (rs2347867) in TS. In combination with previous results, the regression analysis provides good validation of *SOS1* (rs2888586) in GHD and *PTPN1* (rs2038526) in TS.

### The use of random forest classification to investigate the interaction between genetic markers of growth response and patient variables

To further investigate the consistent genetic associations identified using the regression modelling, it was decided to test an independent machine learning-based classification method: random forests. The random forest approach operates by constructing many decision trees to generate an ensemble learning method for classification and is resistant to overfitting and co-linearity.

**Figure 4:**
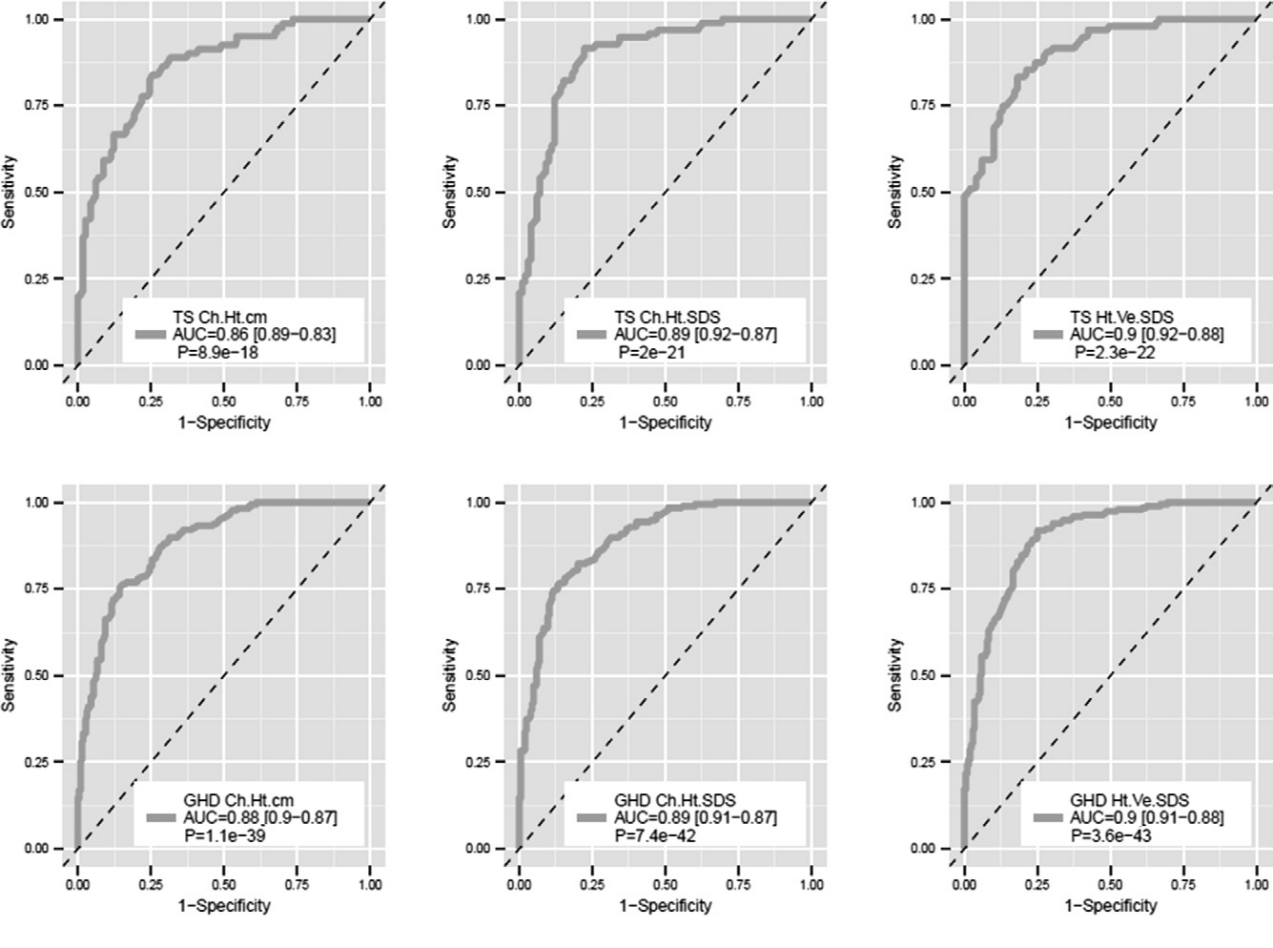
Receiver-operator curve analysis of the random forest modelling of growth response to r-hGH after 1 year of treatment using basal clinical measurements. The receiver-operator curve and associated area under the curve (AUC) are shown for all growth response end points (change in height (cm), change in height SDS and height velocity SDS). A *Z*-score was used to compare the AUC data with the 50% level (dotted line) and used to generate a *P* value.

The set of clinical variables used (gender, age at baseline, GH peak, distance to target height SDS, mid-parental height SDS and GH dose) was shown to be a very good predictor of growth response after 1 year of treatment with r-hGH: Receiver-operator characteristic (ROC) analysis indicated very high levels of sensitivity and specificity (area under the curve (AUC) ~90% in all cases) (Fig. 4 and Table 3A) with an accuracy of 70–80%. Age was found to be an important variable in several classifiers (Table 3A), indicating that the correction of growth response by age category is imperfect.

**Table 3:** Random forest performance (RFP) on auxological parameters, SNPs, auxological parameters and SNPs.

**Disease**	**Endpoint**	**AUC** (95% CI)	**Accuracy**	**Important variables**
(A) RFP on auxological parameters				
GHD	Change in height (cm)	0.88 (0.87–0.90)*	0.80	GH peak, gender
	Change in height SDS	0.89 (0.87–0.90)*	0.80	GH peak, distance to target Ht SDS, age, GH dose, gender
	HV SDS	0.90 (0.88–0.91)*	0.80	GH peak, age, gender, distance to target Ht SDS, GH dose
TS	Change in height (cm)	0.86 (0.84–0.89)*	0.77	Distance to target Ht SDS, MPH SDS, GH dose
	Change in height SDS	0.89 (0.87–0.92)*	0.83	Age, distance to target Ht SDS, GH dose
	HV SDS	0.91 (0.88–0.93)*	0.80	Age, Distance to target Ht SDS
(B) RFP on SNPs				
GHD	Change in height (cm)	0.72 (0.69–0.74)*	0.65	IGF2 (rs3213221)
	Change in height SDS	0.60 (0.57–0.62)*	0.56	IGF2 (rs3213221), SOS1 (rs2888586)
	HV SDS	0.61 (0.58–0.64)*	0.57	HRAS (rs11246176)
TS	Change in height (cm)	0.79 (0.76–0.82)*	0.66	LHX4 (rs4652492), RARB (rs4681028), MYOD1 (rs3911833)
	Change in height SDS	0.58 (0.54–0.62)	0.55	(none)
	HV SDS	0.66 (0.63–0.70)*	0.63	PTPN1 (rs2038526), ESR1 (rs2347887 and rs6927072)
(C) RFP on auxological parameters and SNPs				
GHD	Change in height (cm)	0.88 (0.86–0.90)*	0.80	GH peak, distance to target Ht SDS, GH dose, MPH SDS
	Change in height SDS	0.89 (0.87–0.91)*	0.81	GH peak, age, distance to target Ht SDS, age
	HV SDS	0.86 (0.84–0.88)*	0.77	GH peak, age, MPH SDS, GH dose, distance to target Ht SDS
TS	Change in height (cm)	0.91 (0.88–0.93)*	0.73	LHX4 (rs4652492), RARB (rs4681028), MYOD1 (rs3911833), MPH SDS, GH dose, distance to target Ht SDS, age
	Change in height SDS	0.84 (0.82–0.87)*	0.73	Age
	HV SDS	0.87 (0.84–0.89)*	0.78	Age, PTPN1 (rs2038526), ESR1 (rs2347887 and rs6927072)

* *P* value ≤0.05 (*Z*-test); 95% CI, 95% confidence interval.

SNP-only models using the full range of gene associations identified a number of the SNPs as being significantly important variables, which were acting as weak but distinct predictors with accuracies of 55–66% (Table 3B). *SOS1* identified by the regression analysis was retrieved as an important variable for change in height SDS in the absence of clinical covariates in GHD (Table 3B). ESR1 and PTPN1 SNPs were retrieved among the important variables in TS models of height velocity SDS (Table 3B).

In a full random forest model (all SNPs + all clinical covariates), the two previously identified SNPs (in *PTPN1* and *ESR1*) were also retrieved among the most important variables in TS. In contrast, the two other SNPs (in *INPPL1* and *SOS1*) that were found associated in regression analyses in GHD in interaction with a clinical covariate were not retrieved as important (Table 3C).

### The use of random forest classification in subgroups of GHD severity

Random forest analysis was also used to examine GHD clinical models in the context of GHD severity. GHD models were stratified by GH peak into severe (≤4 μg/L) vs mild (>4 and <10 μg/L) using the merged GHD data.

**Figure 5:**
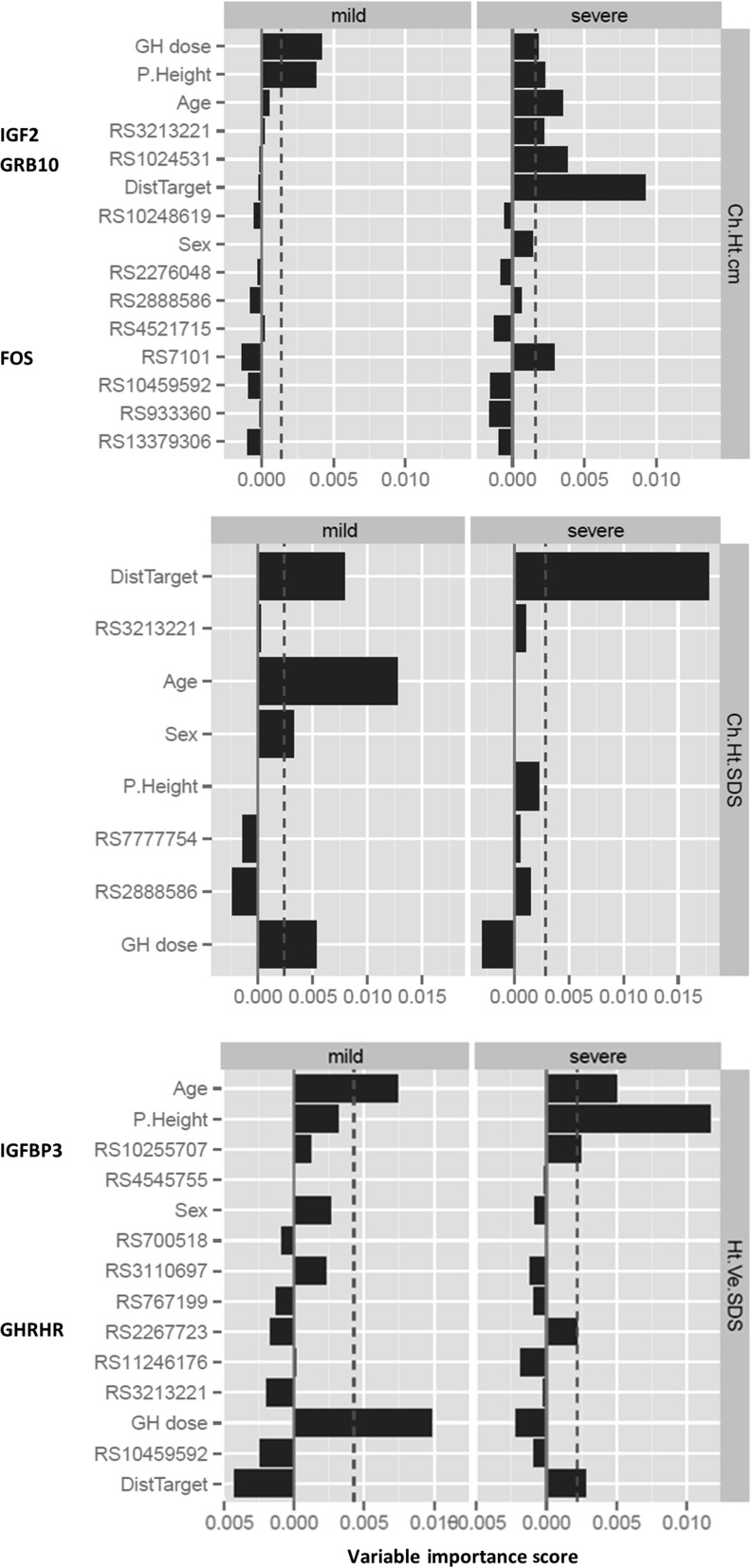
Random forest modelling of growth response to r-hGH after 1 year of treatment in GHD children categorised by GHD severity (severe ≤4 µg/L, mild >4 and <10 µg/L GH peak). Prediction of growth response by random forest analysis in GHD severity-stratified sub-populations. Variable importance scores used to rank variables were derived using the R package random forest. P. Height = mid-parental height SDS, Dist Target = distance to target height SDS.

Prediction of growth response by random forest analysis was similarly good in severity-stratified sub-populations (AUC 85–90%, accuracy 70–75%). Only clinical covariates were important in the mild sub-population, in particular GH dose, age and mid-parental height (Fig. 5). However, in the severe sub-population, there was a contribution of several SNPs identified in the LTFU study (Supplementary Table 1) to the prediction of growth response; the important SNPs were rs3213221 (*IGF2*), rs1024531 (*GRB10*) and rs7101 (*FOS*) in change in height (cm) and rs10255707 (*IGFBP3*) and rs2267723 (*GHRHR*, borderline) in height velocity SDS (Fig. 5).

## Discussion

This study was designed to validate the SNPs identified within the original PREDICT LTFU study as being associated with response to r-hGH therapy in children with either GHD or TS. Although the PREDICT LTFU study was a prospective observational study, the validation study was conducted retrospectively. The main advantage of this retrospective design was that the time taken to collect a second large cohort of children with GHD and TS with data on response to GH therapy was much shorter than would have been required with a second prospective study. Although the inclusion criteria for both the PREDICT LTFU and PREDICT validation studies were identical, the two cohorts of children differed significantly in their baseline auxology. The GHD validation (VAL) cohort was younger at start of GH therapy (by 3.6 years), had a greater mid-parental height SDS and a greater distance to target height (Table 1). The latter was the result of the baseline median height SDS in the VAL cohort being −2.5 and in the LTFU being −2.1 (11). Growth hormone peak level in stimulation testing was however higher in the VAL cohort than that in the LTFU, but the difference was only 1.1 µg/L (Table 1). The patients in TS VAL cohort were also younger (by 3.3 years) and had a greater mid-parental height SDS (Table 1). The baseline median height SDS in the VAL cohort was −2.2 and in the LTFU cohort was −2.4 (11). Both VAL cohorts were treated with lower doses of r-hGH and demonstrated better overall response to treatment compared with the PREDICT LTFU cohort (Table 1).

The reasons for the differences in the cohorts are not clear. All recruits to both studies fulfilled the identical inclusion and exclusion criteria. For the GHD VAL cohort, there may have been an unconscious bias to recruit those with more severe GHD. This would explain the lower age, lower height SDS, greater distance to target height and better response to r-hGH with lower GH doses. It would not explain the higher peak GH levels in the VAL cohort. This observation could be explained however by different GH assays, and the ~1 µg/L difference in peak GH may not be clinically significant. The TS VAL cohort was also younger and responded better to a lower dose of r-hGH but was not shorter than the LTFU cohort. The markedly younger age in the VAL cohorts may be sufficient to explain a better response as age weighs more than GH dose or peak GH when referring to Ranke’s models predictors (4, 6). As both cohorts were recruited from similar centres, it is likely that patients treated in recent years were recruited to the prospective LTFU study, leaving patients treated several years previously to be recruited to the retrospective validation study.

Given the observed differences outlined previously, we needed to use regression analysis with covariates to validate SNPs identified in the PREDICT LTFU study. Using this approach in children with GHD, it was possible to identify SNPs in *INPPL1*, *SOS1*, *GRB10*, *IGFBP3* and *CYP19A1* associated with growth response in all datasets. However, *SOS1* (rs2888586) and *INPPL1* (rs 2276048) were the only two SNPs, which showed consistent effects in both studies. Both encode proteins involved in modulating response to growth hormone/factors (GH for *SOS1* and insulin/IGF1 for *INPPL1*). Both *SOS1* and *INPPL1* are associated with growth disorders – Noonan syndrome (19) and opsismodysplasia (20). It is therefore likely that these SNPs modulate the GH or IGF1 signal transduction pathways either directly or indirectly.

For TS, two SNPs were consistently associated with GH response, these SNPs were in *PTPN1* (rs2038526) and *ESR1* (rs2347867). *ESR1* encodes the oestrogen receptor α, and thus may affect GH response by modulating the actions of oestrogen, which affects GH signal transduction and bone growth (21). PTPN1 encodes a protein tyrosine phosphatase central to growth factor signalling (21).

The *in silico* assessment of SNP variant function identified that rs2038526 within the *PTPN1* gene was located intronically within a MYC transcription factor-binding site, implying possible direct modulation of transcriptional regulation. The other SNPs were either situated in the coding region of the gene but did not alter the amino acid sequence (*INPPL1* (rs 2276048)) or in non-coding regions without evidence of associated transcription factor binding – in the 5′ untranslated region for *SOS1* (rs2888586) and intronically for *ESR1* (rs2347867). It is therefore likely that these SNPs exert their functional influence through more complex genetic mechanisms.

Regression modelling suffers from overfitting and a tendency to underestimate effect sizes if co-linear variables are used. The use of clinical variables alone fails to explain 40–60% of the variability in GH response in established regression models (4, 5). To account for possible overfitting, we have also used a random forest method to predict growth response to r-hGH. The random forest approach operates by constructing multiple decision trees, providing each tree with a random subset of the original data and aggregating the output. Random forests are resistant to overfitting, provide greater accuracy than regression analysis and are efficient with large volumes of data. Using the genomic information alone with a random forest approach, SNPs were identified as important variables, including *SOS1* in the change in height SDS GHD model, strengthening further the validity of SOS1 identified in the regression modelling. These SNPs however predicted response to growth in the first year of treatment with a very modest AUC of 0.58–0.79 (Table 3B). Using the baseline clinical and biochemical data, the random forest gave an AUC of 0.84–0.91 for prediction of growth response in GHD and TS. In addition in TS, *PTPN1* and *ESR1* were identified as important SNPs (Table 3C). In the GHD random forest, SNPs were not found to be important. It is therefore clear that although we can show that genetic variants are associated with GH response, their effects can be attenuated when using a random forest classification. This suggests that in GHD, the effect of SNPs may already be accounted for by variables such mid-parental height SDS and height SDS itself. This was also seen when undertaking a random forest classification (RFC) on the GHD cohort stratified by GHD severity: only clinical variables predicted growth responses in those with mild GHD (Fig. 5). In contrast in those with severe GHD, RFC identified *IGF2, GRB10* and *FOS* (change in height (cms)), and *IGFBP-3* and *GHRHR* (HV SDS) as contributing to growth responses alongside auxological variables, indicating that in these circumstances, the influence of these genes is not co-linear with the auxological variables.

The important question that the PREDICT programme addressed was whether knowledge of an individual genetic background could contribute to prediction of response to r-hGH treatment. Large candidate gene analyses have evolved into genome-wide association studies (GWAS) over the last 10 years, which have demonstrated success at detecting highly significant effects of common gene variants (22), but which have generally failed to explain more than a small amount of the phenotypic variability within datasets (23). Nevertheless, recent analysis has shown that up to 30% of the phenotypic variance in normal adult height can be explained by SNP associations (24). We have now demonstrated that using a candidate gene approach to identify polymorphisms that might relate to growth response to r-hGH we can identify genes in GHD and TS; however, their influence is modest and complicated by interaction with auxological variables that in themselves are related to genetic background. At present, these genes could not be used in a predictive test. We have also shown that random forest classification may be a more robust analytical approach when combining genetic and phenotypic datasets.

Using current technology, a GWAS approach would be preferable and could reveal genes that hitherto have not been implicated in growth mechanisms. An even better approach may be the use of baseline gene expression profiling, which takes a whole genome approach and captures the combined impact of gene and environment on mRNA levels. Using this technique in the PREDICT LTFU cohort, we have previously demonstrated the potential to identify poor responders to r-hGH in GHD and TS (11).

Paediatric pharmacogenomics therefore has the potential to improve the personalisation of medical treatment in growth disorders. The demonstration of clinical utility, however, will require further prospective studies.

## Supplementary data

This is linked to the online version of the paper at http://dx.doi.org/10.1530/EJE-16-0357.

## Declaration of interest

P Chatelain and P Clayton have received research support and honoraria as speakers and consultants from Merck Serono. A Stevens has received honoraria as a speaker from Merck Serono. J Wojcik has received consultation fees from Merck Serono. J Raelson has received consultation fees from Merck Serono. E Koledova is an employee of Merck, Darmstadt, Germany and holds shares in the company. P Murray declares no conflicts.

## Funding

This study was supported by Merck Serono S.A. – Geneva, Switzerland. Additional analysis was supported by Merck, Darmstadt, Germany. Editorial assistance in the form of copy editing, formatting and proofreading was provided by David Candlish, inScience Communications, Chester, UK, funded by Merck, Darmstadt, Germany.

## Author contribution statement

A S developed the analysis and wrote the manuscript. J W performed the analyses. P M, J W, J R, E K and P F contributed to analysis, writing and revision of the manuscript. This work was led by P Ch and P C who oversaw the development of the analyses and manuscript.

## PREDICT investigator group

Argentina: A Belgorosky, Buenos Aires; Australia: G Ambler, Westmead; Austria: K Kapelari, Innsbruck; Canada: C Deal, Montreal; J Hamilton, Toronto; Finland: J Jääskeläinen, Kuopio; France: Y Brusquet, Puyricard; P Chatelain, Lyon; M Colle, Bordeaux; R Coutant, Angers; Y Le Bouc, Paris; R Reynaud, Marseille; J-P Salles, Toulouse; J Weill, Lille. Germany: R Pfäffle, Leipzig; G Binder, Tübingen; Italy: M Bozzola, Pavia; F Buzi, Brescia; M Cappa, Rome; A Cicognani, Bologna; M Maghnie, Genova; F Antoniazzi, Verona. Korea: DH Kim, Seoul; SW Yang, Seoul; HW Yoo, Seoul; Norway: E Vangsøy Hansen, Bergen; D Veimo, Bodø; Russia: E Bashnina, St Petersburg; V Peterkova, Moscow; J Skorodok, St Petersburg; L Sultanova, Kazan; Spain: A Carrascosa, Barcelona; A Ferrandez Longas, Zaragoza; R Gracia Bouthellier, Madrid; JP Lopez Siguero, Malaga; S Quinteiro, Las Palmas de Gran Canaria; MD Rodriguez-Arnao, Madrid; A Rodriguez Sanchez, Madrid; Sweden: J Dahlgren, Göteborg; L Hagenäs, Stockholm; Taiwan: JW Hou, Taoyuan; TJ Wang, Kaohsiung County; UK: P Clayton, Manchester; C Kelnar, Edinburgh.
